# The association of highly processed food consumption with food choice values and food literacy in Japanese adults: a nationwide cross-sectional study

**DOI:** 10.1186/s12966-023-01538-7

**Published:** 2023-12-05

**Authors:** Nana Shinozaki, Kentaro Murakami, Xiaoyi Yuan, Ryoko Tajima, Mai Matsumoto, Keiko Asakura, Shizuko Masayasu, Satoshi Sasaki

**Affiliations:** 1 Department of Nutritional Epidemiology and Behavioural Nutrition, Graduate School of Medicine, The University of Tokyo, 7-3-1 Hongo, Bunkyo-ku, Tokyo 113-0033 Japan; 2 Department of Social and Preventive Epidemiology, School of Public Health, The University of Tokyo, 7-3-1 Hongo, Bunkyo-ku, Tokyo 113-0033 Japan; 3 Department of Nutritional Epidemiology and Shokuiku, National Institute of Biomedical Innovation, Health and Nutrition, Kento Innovation Park, NK Building, 3-17 Senrioka Shinmachi, Settsu-shi, Osaka 566-0002 Japan; 4 Department of Preventive Medicine, School of Medicine, Toho University, 5-21-16 Omori-Nishi, Ota-ku, Tokyo 143-8540 Japan; 5 Ikurien-Naka, 3799-6 Sugaya, Naka-shi, Ibaraki 311–0105 Japan

**Keywords:** Highly processed foods, Ultra-processed foods, Food choice values, Food literacy, Japan

## Abstract

**Background:**

Highly processed food (HPF) consumption is increasing globally and has become a prominent public health concern. However, the relationship between HPF consumption and food choice values and food literacy is unknown. This study aimed to examine the association of HPF consumption with food choice values and food literacy.

**Methods:**

This cross-sectional study used data from a nationwide questionnaire survey conducted in 2018 among 2232 Japanese adults aged 18–80 years. We assessed eight food choice values (accessibility, convenience, health/weight control, tradition, sensory appeal, organic, comfort, and safety) using a 25-item scale, and food literacy characterised by nutrition knowledge (using a validated 143-item questionnaire), cooking and food skills (using 14- and 19-item scales, respectively), and eight eating behaviours (hunger, food responsiveness, emotional overeating, enjoyment of food, satiety responsiveness, emotional undereating, food fussiness, and slowness in eating, using the 35-item Adult Eating Behavior Questionnaire). HPF consumption was estimated using a validated brief diet history questionnaire. The associations between HPF consumption and age, body mass index, energy intake, and each score on food choice values and food literacy were evaluated by multiple linear regression.

**Results:**

In males, one standard deviation increase in scores for cooking skill and satiety responsiveness was associated with an increase in HPF consumption by 22.1 g/4184 kJ (95% confidence interval (CI): 6.6 to 37.5) and 15.4 g/4184 kJ (95% CI: 6.0 to 24.7), respectively. In females, one standard deviation increase in age and scores for safety and nutrition knowledge corresponded to a decrease in HPF consumption by − 16.4 g/4184 kJ (95% CI: − 23.4 to − 9.3), − 9.9 g/4184 kJ (95% CI: − 19.1 to − 0.7), and − 11.1 g/4184 kJ (95% CI: − 17.0 to − 5.3), whereas one standard deviation increase in the satiety responsiveness score corresponded to an increase in HPF consumption by 13.1 g/4184 kJ (95% CI: 6.8 to 19.4).

**Conclusions:**

This cross-sectional study suggests that several aspects of food choice values and food literacy were associated with HPF consumption in Japanese adults. Further studies are needed to confirm our findings in a broader context.

**Supplementary Information:**

The online version contains supplementary material available at 10.1186/s12966-023-01538-7.

## Background

Highly processed foods (HPFs) are defined as multi-ingredient industrially formulated mixtures [1], such as sugar-sweetened beverages and confectioneries. Dramatic increases in HPF consumption have been reported worldwide [2, 3]. Previous studies have suggested that increased HPF consumption was associated with low overall diet quality, characterised by a high intake of total fats, saturated fats, trans fats, and added and free sugars, as well as a lower intake of vegetables, fibre, vitamins (e.g., vitamins A, C, and D), and minerals (e.g., potassium and iron) [4–9]. Moreover, meta-analyses have shown that high HPF consumption is a potential risk factor for overweight and obesity, cardiovascular and cerebrovascular disease, metabolic syndrome, depression, and mortality [10–12]. Currently, several countries recommend reducing HPF consumption in their official dietary guidelines, including Brazil [13], Canada [14], New Zealand [15], and Israel [16].

However, encouraging behavioural change towards healthy eating is not straightforward and requires a better understanding of conditions preceding behaviour, such as the determinants of food choice [17, 18]. Individual food choice is largely influenced by a variety of structural factors, such as food-related features (e.g., colour, aroma), person-related factors (e.g., biological features, cognitive factors), sociocultural factors (e.g., culture, regulations), and food environment (e.g., food availability, marketing) [19, 20]. Among person-related factors, researchers are increasingly incorporating the concept of food choice values, defined as ‘factors that individuals consider when deciding which foods to purchase and/or consume’ [21]. Food choice values are considered proximal influences on food choice that convey the influence of more distant determinants, such as food environment [21]. Furthermore, making healthy food choices is influenced by food literacy, defined as ‘a collection of inter-related knowledge, skills and behaviours required to plan, manage, select, prepare and eat food to meet needs and determine intake’ [22]. Today, HPFs dominate the food system due to their palatability, availability, affordability, and market strategies, especially in high-income countries [2, 4, 19, 20]. In the context where people are routinely exposed to HPFs through shopping and advertising, understanding how food choice values and food literacy are associated with HPF consumption is important to promote healthier food choices for individuals [20, 21]. However, there is a paucity of research on the relationship between HPF consumption and food choice values or food literacy [23–25]. Only a few qualitative studies have identified some personal factors that may influence HPF consumption, such as cooking skills, health consciousness, sustainability awareness, time and financial constraints, preferences, and values of availability and convenience [26–28]. Moreover, the types of HPFs investigated in previous studies were limited to several specific food items, such as convenience food [23, 24]. Given the complexity and multifaceted nature of food choices [20] and the impact of HPF consumption on public health [10–12], a comprehensive assessment of individual internal factors such as food choice values and food literacy related to total HPF consumption is necessary.

This study aimed to examine the association of HPF consumption with food choice values and food literacy. We have characterised food literacy as nutrition knowledge, cooking and food skills, and eating behaviours, based on the most widely used description of food literacy [29].

## Methods

### Study participants and procedure

This cross-sectional study was based on a nationwide questionnaire survey conducted between October and December 2018. Details of the survey are provided elsewhere [30, 31]. Briefly, 422 local research dietitians recruited healthy adults aged 18–80 years residing in 32 of the 47 prefectures throughout Japan. Participants were selected to include an approximately equal number of participants for each sex and age group (18–29, 30–39, 40–49, 50–59, 60–69, and 70–80 years). The inclusion criteria were free-living adults willing to participate in the study. Exclusion criteria were dietitians, those living with a dietitian, working with a research dietitian, those who had received dietary counselling from a doctor or dietitian, those receiving insulin or dialysis treatment, and pregnant or lactating women. Only one individual per household was allowed to participate. In total, 2247 adults participated in this survey.

Participants were asked to complete a brief diet history questionnaire (BDHQ) and four questionnaires on food choice values, nutrition knowledge, cooking and food skills, and eating behaviours, as described later. Responses to the BDHQ were thoroughly checked by research dietitians and then by the corresponding author, and those to the other questionnaires (except for nutrition knowledge) were reviewed by research centre staff. If any responses were missing or erroneous, the participant was asked to answer the question again in person or by telephone. We excluded participants who lacked information on any of the variables of interest (*N* = 3) and those aged over 80 years (*N* = 12), leaving 2232 participants aged 19–80 years; 361 aged 18–29 years, 345 aged 30–39 years, 375 aged 40–49 years, 377 aged 50–59 years, 376 aged 60–69 years, and 398 aged ≥ 70 years.

### Dietary assessment

#### Brief diet history questionnaire

Dietary intake was assessed with the BDHQ. Details of the BDHQ have been published elsewhere [32, 33]. Briefly, the BDHQ is a four-page self-administered questionnaire on dietary habits in the previous month. It includes structured questions about the consumption frequency of commonly consumed foods and general dietary behaviours. Estimates of daily intakes of food groups, energy, and nutrients were calculated using a custom computer algorithm for the BDHQ. The algorithm incorporates sex-specific portion sizes primarily determined based on Japanese cookbooks and the nutrient composition of each food item derived from the Standard Tables of Food Composition in Japan [34]. The validity of the BDHQ has been examined in 92 females and 92 males, using a 16-day weighed dietary record (DR) as reference [32, 33]. Briefly, the median Spearman correlation coefficient for food groups was 0.44 (range: 0.14–0.82) in females and 0.48 (range: 0.22–0.83) in males [32], while the median Pearson correlation coefficient for nutrients was 0.54 (range: 0.27–0.84) in females and 0.56 (range: 0.19–0.81) in males [31]. The BDHQ also included questions about the participant’s sex, age, body height, and weight. Body mass index (BMI) was calculated as self-reported body weight (kg) divided by self-reported height squared (m^2^).

#### Calculation of highly processed food consumption

In this study, HPF was defined as multi-ingredient industrially formulated mixtures processed to the extent that they are no longer recognisable as their original plant or animal source, such as margarine, sausage, processed cheese, and frozen pizza [1]. HPF consumption was calculated based on responses to the BDHQ, which uses 147 food codes (Table S1) to compute dietary intake. Generally, HPF consumption based on diet questionnaires is calculated by classifying each food item in the questionnaires as HPF or non-HPF [35, 36]. However, this procedure may cause misestimation of food items because some of the food groups in the BDHQ consist of several different food codes, each of which may be classified as HPF or non-HPF according to preparation and processing methods (e.g., carrot in a packaged ready-to-eat curry is classified as HPF while carrot in a home-made curry is classified as non-HPF). Therefore, we estimated the probability of including HPF for each food code used in the BDHQ and then used the estimated probability to calculate the HPF intake from the BDHQ.

The probability of including HPF was determined for each food code as a weight ratio of HPFs to total intake, using DR data (comprising 1568 days of data) with a detailed classification of HPF previously obtained from another Japanese population. The DR data were 4-day DRs obtained from 392 Japanese adults aged 20–69 years in 2013, who also completed the BDHQ prior to conducting the DR. Details of the survey have been provided elsewhere [37]. Each food item excluding dietary supplements in the DR was assigned a food code from the Standard Tables of Food Composition in Japan [34]. Then, food items were classified into one of four groups: unprocessed and minimally processed, basic processed, moderately processed, and highly processed, based on the food classification system developed by the University of North Carolina at Chapel Hill (UNC) [1] using a stepwise classification procedure (Figure S1). The food categories of the UNC system are consistent with those of NOVA [38]; ‘highly processed’ in the UNC system is equivalent to ‘ultra-processed’ in NOVA [1].

Participants in this DR data set were randomised into calculation (*N* = 199) or validation (*N* = 193) groups (Table S2). Using the DR data in the calculation group, the weight ratio of HPF was calculated for each food code in the BDHQ as follows: weight ratio of HPF for a food code = the sum of foods identified as ‘highly processed’ in the food code (g) / total food consumption in the food code (g) × 100 (Table S1). The calculated weight ratio of HPF for each food code was then used to estimate HPF consumption from the BDHQ in the validation group, as follows. The BDHQ computes the intake of each food group (*N* = 58) as the sum of the weight of different food items included in the food group. For instance, the ‘mayonnaise and dressing’ group intake is calculated as the sum of the weights of ‘mayonnaise’ (food code 17043) and ‘French dressing’ (food code 17040). Therefore, we first calculated the estimated HPF consumption from each food item (e.g., mayonnaise) for each participant as the total intake of the food item (g) multiplied by its weight ratio of HPF. Subsequently, the HPF consumption from each food group (e.g., ‘mayonnaise and dressing’ group) was calculated by summing the HPF consumption from each food item within that food group (e.g., the mayonnaise and French dressing group). Finally, the total HPF consumption per person was computed as the sum of the HPF intake from all 58 food groups.

The validity of HPF consumption estimated based on the BDHQ was assessed in the validation group using HPF consumption estimated from the DR as a reference. The results showed that the Spearman correlation coefficient for HPF consumption (g per 4184 kJ) was 0.59 for males (*N* = 97) and 0.46 for females (*N* = 96), indicating moderate correlation. The limits of agreement were wide for both sexes, mainly because of increased dispersion with larger HPF consumption (Figure S2). Further details of the calculation and validation of HPF consumption based on the BDHQ are described in Text S1.

Using the weight ratio of HPF for each food code, we estimated the HPF intake (g/day) for each participant in the present questionnaire survey from the BDHQ, using the same calculation process as described for the validation group. Daily HPF consumption was adjusted using the nutrient density method and expressed as grams per 4184 kJ (1000 kcal) to correct potential measurement errors [39].

### Food choice values

Food choice values were assessed by the Japanese version of the food choice value questionnaire. Details of the structure, validity, and reliability validity of the original English version of the questionnaire [21] and the development process of the Japanese version [30] have been described elsewhere. The food choice value questionnaire is a 25-item, self-administered questionnaire measuring eight components of food choice values: accessibility, convenience, health/weight control, tradition, sensory appeal, organic, comfort, and safety [21]. Participants were asked, ‘When deciding what foods to buy or eat on a daily basis, how important are each of the following?’ The possible responses were on a Likert scale ranging from 1 to 5 (1: not at all, 2: a little, 3: moderately, 4: quite a bit, and 5: very). The score for each factor was calculated by dividing the sum of the scores by the number of items (4 items for organic and 3 items for the other factors), with possible scores ranging from 1 to 5.

### Food literacy

#### Nutrition knowledge

Nutrition knowledge was assessed using the Japanese general nutrition knowledge questionnaire (JGNKQ). Details of the structure, validity, and reliability of the JGNKQ are available elsewhere [40]. The JGNKQ is a self-administered questionnaire originally consisting of a 147-item in 5 sections (dietary recommendations, sources of nutrients, choosing everyday foods, diet-disease relationships, and reading a food label). This study used a 143-item version of the JGNKQ, with a very low percentage of correct responses removed from the original version [30]. For each item, one point was given for a correct response and zero for an incorrect or missing response. Thus, the possible scores ranged from 0 to 143, with a higher score indicating a higher level of nutrition knowledge.

#### Cooking and food skills

Cooking and food skills were assessed by the Japanese version of the English scale for cooking and food skills. Details of the structure, validity, and reliability of the original English version of the scale [41] and the development process of the Japanese version [30] have been provided elsewhere. Questions on cooking skills (14 items) ask about cooking methods and food preparation techniques, whereas those on food skills (19 items) ask about meal planning and preparing, shopping, budgeting, resourcefulness, and label reading/consumer awareness. Participants were asked to rate how good they were at each skill on a 7-point Likert scale from 1 (very poor) to 7 (very good). If the participant did not use a skill, they could choose an option of ‘never/rarely do it’, to which a score of zero was assigned. The scores of cooking and food skills were calculated as the sum of all the items, with possible scores ranging from 0 to 98 for cooking skill and from 0 to 133 for food skill.

#### Eating behaviours

Eating behaviours were assessed by the Japanese version of the Adult Eating Behaviour Questionnaire (AEBQ). Details of the structure, validity, and reliability validity of the original English version [42] and the development process of the Japanese version [30] have been described elsewhere. The AEBQ is a 35-item, self-administered questionnaire measuring four food approach scales (hunger, food responsiveness, emotional overeating, and enjoyment of food) and four food avoidance scales (satiety responsiveness, emotional undereating, food fussiness, and slowness in eating) [42]. Responses were rated based on a 5-point Likert scale for each behaviour ranging from ‘strongly disagree’ to ‘strongly agree,’ and a mean score of 1 to 5 was calculated across all scales.

### Statistical analysis

All analyses were performed separately for males and females. This is because the distribution of food choice values, nutrition knowledge, cooking and food skills, and eating behaviours differ markedly between sexes [30] and because prior analyses indicated a suggestion of heterogeneity by sex in the association of HPF consumption with age and cooking skills (p for interaction = 0.02 and 0.047, respectively). Data are shown as means and standard deviations (SDs). The difference between males and females was tested using the Mann–Whitney U test. Univariate linear regression analyses were performed to assess the association of HPF consumption (in grams per 4184 kJ) with independent variables: age, BMI, energy intake (in kJ per day), and each score for food choice values, nutrition knowledge, cooking and food skills, and eating behaviours (all treated as continuous variables). Results are presented as β coefficients and 95% confidence interval (CI), which mean a change in HPF consumption (g per 4184 kJ) per 1-SD increase in each independent variable. Multivariable analyses were used to assess the independent effects of variables on HPF consumption; all variables were entered simultaneously into the model. Multicollinearity among covariates in the multivariable-adjusted model was assessed using variance inflation factors [43]. All variance inflation factors were less than 4, indicating low collinearity. All statistical analyses were performed using Statistical Analysis System (SAS) version 9.4 (SAS Institute Inc., Cary, NC, USA). Two-sided *P*-values < 0.05 were considered statistically significant.

## Results

A total of 2232 participants (1069 males and 1163 females) aged 19–80 years were included in the analysis. Table 1 shows the participant characteristics. The mean age was 50.3 years (SD: 17.2) in males and 50.0 years (SD: 17.5) in females. Females had significantly lower HPF consumption than males (*P* < 0.0001). For food choice values, the highest mean score was observed in sensory appeal (3.2 points) in males and safety (3.5 points) in females. Females had significantly higher scores in nutrition knowledge, cooking skills, and food skills (*P* < 0.0001). Among eating behaviours, the highest mean score was observed in enjoyment of food in both males and females (3.9 points and 4.1 points, respectively).

**Table 1:** Participant characteristics

**Characteristic**	**All**		**Male**		**Female**	
	**(*N* = 2232)**		**(*N* = 1069)**		**(*N* = 1163)**	
	**Mean**	**SD**	**Mean**	**SD**	**Mean**	**SD**
Age (years)	50.2	17.3	50.3	17.2	50.0	17.5
Body height (cm)	162.6	8.9	169.4	6.3	156.3	5.9
Body weight (kg)	60.9	12.1	68.0	10.9	54.4	9.0
Body mass index (kg/m^2^)	22.9	3.5	23.7	3.3	22.3	3.5
Energy intake (kJ/day)	7694	2222	8518	2307	6938	1842
HPF consumption (g/4184 kJ)	194	124	222	141	169	99
Food choice values (1–5)^a^						
Accessibility	3.2	0.8	3.1	0.8	3.3	0.7
Convenience	3.1	0.8	2.9	0.9	3.2	0.8
Health/weight control	2.8	0.9	2.7	1.0	3.0	0.8
Tradition	2.1	0.8	2.0	0.8	2.2	0.7
Sensory appeal	3.3	0.7	3.2	0.7	3.4	0.7
Organic	2.9	0.8	2.7	0.9	3.2	0.8
Comfort	2.3	0.8	2.2	0.8	2.5	0.8
Safety	3.3	0.9	3.1	0.9	3.5	0.8
Nutrition knowledge (1–143)^a^	70.2	24.6	63.9	25.9	76.0	21.8
Cooking skills (0–98)^a^	43.3	26.0	30.3	25.9	55.2	19.5
Food skills (0–133)^a^	62.5	34.6	43.6	34.1	79.9	24.4
Eating behaviours (1–5)^a^						
Hunger	2.8	0.7	2.7	0.7	2.9	0.7
Food responsiveness	2.7	0.7	2.6	0.6	2.9	0.7
Emotional overeating	2.4	0.8	2.2	0.8	2.5	0.8
Enjoyment of food	4.0	0.7	3.9	0.8	4.1	0.7
Satiety responsiveness	2.6	0.7	2.5	0.7	2.7	0.7
Emotional undereating	2.7	0.9	2.6	0.9	2.9	0.8
Food fussiness	2.6	0.8	2.6	0.8	2.5	0.8
Slowness in eating	2.6	0.7	2.4	0.7	2.7	0.7

*HPF* highly processed food, *SD *standard deviation

^a^The range of possible scores is shown in the parenthesis

The associations of HPF consumption with food choice values and food literacy variables among males are shown in Table 2. In unadjusted models, HPF consumption (g) per 4184 kJ was positively associated with cooking skills, food skills, and satiety responsiveness and inversely associated with hunger. After adjusting for all variables in the model, significant associations remained only for cooking skills and satiety responsiveness. Each 1-SD (25.9 points) increment in cooking skill score corresponded to a 22.1 g per 4184 kJ (95% CI: 6.6 to 37.5) increase in HPF consumption. Similarly, a 1-SD (0.7 points) increase in satiety responsiveness score was associated with a 15.4 g per 4184 kJ (95% CI: 6.0 to 24.7) increase in HPF consumption.

**Table 2:** HPF consumption and food choice values and food literacy variables in males (*N* = 1069)

Variable	**Unadjusted**^**a**^				**Adjusted**^**b**^			
	**Regression coefficient**^**c**^	**95% confidence interval**		***p*-value**	**Regression coefficient**^**c**^	**95% confidence interval**		***p*-value**
		**Lower**	**Upper**			**Lower**	**Upper**	
Age (years)	-6.6	-15.0	1.8	0.13	-2.2	-12.1	7.6	0.66
Body mass index (kg/m^2^)	-2.5	-11.0	5.9	0.56	0.9	-8.2	10.0	0.85
Energy intake (kJ/day)	0.2	-8.2	8.7	0.96	3.6	-5.3	12.4	0.43
Food choice values (1–5)^d^								
Accessibility	-1.6	-10.1	6.8	0.70	-4.9	-16.5	6.7	0.41
Convenience	1.7	-6.8	10.1	0.70	6.4	-5.0	17.8	0.27
Health/weight control	-7.6	-16.0	0.8	0.08	-6.2	-17.7	5.3	0.29
Tradition	-5.6	-14.1	2.8	0.19	-8.0	-18.7	2.7	0.14
Sensory appeal	1.9	-6.5	10.4	0.66	6.2	-4.5	16.9	0.26
Organic	-7.9	-16.3	0.5	0.07	-5.9	-22.4	10.6	0.49
Comfort	2.5	-6.0	10.9	0.57	10.9	-0.4	22.2	0.06
Safety	-7.8	-16.2	0.7	0.07	-4.5	-19.7	10.6	0.56
Nutrition knowledge (1–143)^d^	0.2	-8.2	8.7	0.96	-1.8	-11.0	7.3	0.69
Cooking skills (0–98)^d^	15.0	6.6	23.4	0.0005	22.1	6.6	37.5	0.005
Food skills (0–133)^d^	8.6	0.2	17.1	0.04	-5.9	-21.9	10.2	0.47
Eating behaviours (1–5)^d^								
Hunger	-9.0	-17.4	-0.6	0.04	-6.8	-17.1	3.5	0.19
Food responsiveness	-8.2	-16.6	0.2	0.06	-5.0	-16.2	6.2	0.38
Emotional overeating	-7.8	-16.2	0.7	0.07	-4.2	-14.2	5.7	0.41
Enjoyment of food	-5.8	-14.2	2.7	0.18	-2.2	-12.2	7.9	0.67
Satiety responsiveness	12.7	4.3	21.2	0.003	15.4	6.0	24.7	0.001
Emotional undereating	-4.6	-13.1	3.8	0.28	-4.1	-13.3	5.1	0.38
Food fussiness	1.0	-7.5	9.5	0.82	-1.2	-10.9	8.4	0.80
Slowness in eating	-1.4	-9.8	7.1	0.75	-2.2	-11.5	7.1	0.64
HPF, highly processed food								

^a^Univariate model

^b^Multivariable model. All variables were entered into the model simultaneously

^c^Regression coefficients mean the change of consumption of highly processed foods (g/4184 kJ) with one standard deviation increase in each variable

^d^The range of possible scores is shown in the parenthesis

The results of the same analysis for females are shown in Table 3. In unadjusted models, HPF consumption was positively associated with food responsiveness and satiety responsiveness and negatively associated with age, food choice values for organic and safety, nutrition knowledge, and food skills. After adjustment for all variables, the association remained significant for age, safety, nutrition knowledge, and satiety responsiveness. Each 1-SD increase in age (SD: 17.5), safety (SD: 0.8), and nutrition knowledge (SD: 21.8) was equivalent to a decrease in HPF consumption by − 16.4 g/4184 kJ (95% CI: − 23.4 to − 9.3), − 9.9 g/4184 kJ (95% CI: − 19.1 to − 0.7), and − 11.1 g/4184 kJ (95% CI: − 17.0 to − 5.3). Meanwhile, a 1-SD increase in the satiety responsiveness score (SD:0.7) corresponded to an increase in HPF consumption by 13.1 g/4184 kJ (95% CI: 6.8 to 19.4).

**Table 3:** HPF consumption and food choice values and food literacy variables in females (*N* = 1163)

Variable	**Unadjusted**^**a**^				**Adjusted**^**b**^			
	**Regression coefficient**^**c**^	**95% confidence interval**		***p*-value**	**Regression coefficient**^**c**^	**95% confidence interval**		***p*-value**
		**Lower**	**Upper**			**Lower**	**Upper**	
Age (years)	-18.5	-24.1	-12.9	< 0.0001	-16.4	-23.4	-9.3	< 0.0001
Body mass index (kg/m^2^)	-5.5	-11.2	0.2	0.06	-4.9	-11.0	1.2	0.12
Energy intake (kJ/day)	-4.1	-9.8	1.6	0.16	3.4	-2.5	9.3	0.26
Food choice values (1–5)^d^								
Accessibility	0.9	-4.8	6.6	0.76	2.8	-4.0	9.6	0.42
Convenience	0.0	-5.7	5.7	0.996	-3.7	-10.4	3.0	0.28
Health/weight control	0.0	-5.7	5.8	0.99	5.7	-1.3	12.7	0.11
Tradition	-4.7	-10.4	1.0	0.11	0.9	-6.4	8.1	0.81
Sensory appeal	0.9	-4.8	6.6	0.76	2.2	-4.7	9.2	0.53
Organic	-13.0	-18.6	-7.3	< 0.0001	-4.5	-14.5	5.5	0.38
Comfort	0.6	-5.1	6.3	0.83	2.0	-5.3	9.3	0.59
Safety	-13.6	-19.3	-8.0	< 0.0001	-9.9	-19.1	-0.7	0.03
Nutrition knowledge (1–143)^d^	-9.7	-15.4	-4.0	0.0008	-11.1	-17.0	-5.3	0.0002
Cooking skills (0–98)^d^	-3.4	-9.1	2.3	0.24	6.4	-2.0	14.7	0.14
Food skills (0–133)^d^	-6.7	-12.4	-1.0	0.02	-3.0	-11.5	5.5	0.50
Eating behaviours (1–5)^d^								
Hunger	5.2	-0.5	10.9	0.08	-3.2	-10.2	3.9	0.38
Food responsiveness	7.7	2.0	13.4	0.008	3.2	-4.6	10.9	0.43
Emotional overeating	5.4	-0.3	11.1	0.06	2.8	-4.0	9.5	0.42
Enjoyment of food	0.6	-5.1	6.3	0.83	-1.8	-8.7	5.2	0.62
Satiety responsiveness	10.7	5.0	16.3	0.0002	13.1	6.8	19.4	< 0.0001
Emotional undereating	-1.3	-7.0	4.4	0.66	-3.9	-9.9	2.1	0.20
Food fussiness	2.6	-3.1	8.4	0.36	-2.1	-8.4	4.2	0.51
Slowness in eating	-1.6	-7.3	4.1	0.58	-3.0	-8.8	2.8	0.31
HPF, highly processed food								

^a^Univariate model

^b^Multivariable model. All variables were entered into the model simultaneously

^c^Regression coefficients mean the change of consumption of highly processed foods (g/4184 kJ) with one standard deviation increase in each variable

^d^The range of possible scores is shown in the parenthesis

## Discussion

We performed a cross-sectional examination of the association between HPF consumption and food literacy (nutrition knowledge, cooking and food skills, and eating behaviours) and food choice values. In males, HPF consumption was positively associated with cooking skills and satiety responsiveness. In females, HPF consumption was positively associated with satiety responsiveness while inversely associated with age, safety, and nutrition knowledge. To the best of our knowledge, this is the first study to comprehensively investigate the association of HPF consumption with food choice values and food literacy.

Both HPF and ultra-processed food refer to foods in the highest category of processing in various food classification systems [44], and research on HPF/ ultra-processed food is spreading worldwide [1–12]. However, only a few studies have been conducted on the association between HPF consumption and personal factors such as food choice values and food literacy. For example, in a study of German adults (*N* = 814), the consumption frequency of some HPFs, such as plant-based meat alternatives, was associated with different attitudinal and behavioural factors, including cooking frequency and sustainable food choice motives [24]. In addition, in a sample of the Swiss adult population (*N* = 918), the consumption frequency of highly-processed convenience food items (e.g., ready meals) was inversely associated with sociability (enjoying eating with others), concerns of naturalness, nutritional knowledge, and cooking skills [23]. However, these studies investigated a limited number of food items using food frequency questionnaires with unknown validity and did not comprehensively investigate food choice values or food literacy.

In terms of food choice values, we found that HPF consumption in females was inversely associated with safety, defined as ‘the degree to which food has been prepared or processed properly and will not cause illness’ [21]. The safety and healthfulness of HPF have been identified as important factors that consumers consider when purchasing or consuming HPF [26, 28]. Thus, females who value food safety would be concerned about the safety and healthfulness of HPFs and may avoid them. We also observed that nutrition knowledge was inversely associated with HPF consumption in females, consistent with a previous study [23]. The nutrition knowledge questionnaire used in this study did not specifically ask about food processing or HPFs, whereas it included many questions about the nutrient content of common foods. Given that HPFs generally have less favourable nutrient profiles [4–9], it is not surprising that a better understanding of nutrients results in less selection of HPFs. Meanwhile, no association was observed between HPF consumption and the food choice value of safety or nutrition knowledge in males. This may be due to a gender difference in involvement in purchasing and cooking. That is, since males tend to be less responsible for buying and preparing foods than females in Japan [45], the food choice value of safety and nutritional knowledge among males may not directly influence their HPF consumption.

Previous studies have reported inverse associations between cooking skills and HPF consumption [23, 46]. By contrast, we observed that males with lower cooking skills had lower HPF consumption. Although the reason for this is unclear, there may be the possibility of residual confounding by marital status. For example, Japanese males with lower cooking skills tended to be married, have a family member as the main meal preparer, and have a lower frequency of home cooking, while most Japanese females cooked by themselves [45]. Moreover, in Portugal, married males had a lower HPF consumption than single males [47]. Thus, participants with lower cooking skills may be more likely to be married and have lower HPF consumption because their dietary intake was dominated by the cohabiting female with lower HPF consumption. However, we could not examine the potential impact of marital status since the information on marital status was unavailable in this study. Therefore, further research would be needed to clarify this aspect of the association between cooking skills and HPF consumption.

A previous study has reported that higher HPF consumption at age 4 years was directly associated with food responsiveness (i.e., eating in response to external food cues) and indirectly through energy intake with food fussiness (i.e., a lack of interest in food and unwillingness to try new foods) and satiety responsiveness (i.e., the ability to regulate the amount of food eaten, based on perceived fullness) at age 7 years [25]. However, there has been little evidence on the association between HPF consumption and eating behaviours among adults. In this study, both male and female participants with higher satiety responsiveness had higher HPF consumption. The naïve interpretation of this association would be that adults who tend to feel full easily would eat more HPFs. However, caution should be exercised in interpretation due to possible measurement errors and confounding. For example, we did not directly assess satiety responsiveness, such as using appetite rating or physiological measurement at each meal [48]. Moreover, while HPF is reportedly energy-dense and less satiating [49], satiety is influenced not only by the satiating potential of food but also by various internal and external factors in humans, such as age, gender, and eating with others [48]. Furthermore, satiety responsiveness develops in the early stages of life and is influenced by genetic and environmental factors [50], which could confound our results. Therefore, further studies are needed to clarify the association between HPF consumption and eating behaviours, including the satiety responsiveness of individuals.

Changes in age would affect physiological, metabolic and psychological responses to food, possibly impacting food choice [51]. In this study, age was inversely associated with HPF intake in females, consistent with previous studies [5, 7, 47]. This may be because younger adults tend to consume more food outside the home and at work and spend less time cooking, while older adults spend more time cooking and are less familiar with convenience products [23, 25]. Moreover, given that socioeconomic position, such as income, is associated with age [52] and HPF consumption [53], the association between age and HPF consumption may be mediated by socioeconomic factors, unavailable in this study.

Some strengths of this study are that it targeted a large nationwide sample, including approximately equal proportions of males and females in a wide range of age groups from diverse regions throughout Japan. In addition, we comprehensively assessed food choice values and food literacy (nutrition knowledge, cooking and food skills, and eating behaviours) using well-established scales and evaluated their associations with HPF consumption, controlling for the potential confounding effects of each parameter on the others. Nevertheless, some limitations warrant mention. First, since study participants were not randomly selected but were healthy volunteers willing to participate, they may be more health conscious than the general population. However, the mean (SD) height, weight, and BMI of the study participants were similar to those of a national representative sample aged ≥ 20 years (males: 168.0 (7.1) cm, 67.3 (11.1) kg, and 23.8 (3.7) kg/m^2^, respectively; females: 154.5 (7.0) cm, 53.5 (9.3) kg, and 22.5 (3.7) kg/m^2^, respectively) [54]. Thus, there may be no strong reason to believe that the participants in this study are greatly different from the general Japanese population. Second, we could not fully examine possible internal and external factors associated with HPF consumption due to a lack of information. For instance, previous studies have shown that higher HPF consumption was associated with marital status [47], smoking [49], sedentary behaviour [55], eating location [56] and lower availability of food retailers [57]. Thus, there may be the possibility of residual confounding from socioeconomic, behavioural, and environmental factors. Although information on various basic characteristics had been collected on this population in the past, the data usage has not been approved by the Ministry of Health, Labour and Welfare [58]. However, a previous study of 1165 Japanese adults aged 18–64 reported no association between nutrition knowledge and educational status or household income [59]. Given that food choices are influenced by various structural factors [18, 20], future studies should include other possible facilitators of HPF consumption, such as part-time work, non-employment, lower sociability, bigger family size, and longer screen time [23, 60]. Third, the questionnaires for food choice values, cooking and food skills, and eating behaviour were originally developed and validated in Western countries. To increase comparability between the Japanese and English versions, the Japanese version was developed without considering the cultural differences between countries. Thus, the questionnaires may not be well suited to the Japanese population. However, the internal consistency of all scores (except for slowness in eating) was comparable to that observed in previous studies [30]. Fourth, the BDHQ was not developed to collect information on food processing, which may not be optimal for capturing HPF consumption. Moreover, the classification of HPF in the four-day DR was performed by a single author. Although the classification system used in the present study was reported to have high reliability between those rating responses [44], some foods may have been misclassified, possibly resulting in a misestimation of the weight ratio of HPF. In addition, the calculation and validation of the HPF weight ratios were conducted in two randomly assigned groups from the same population and the sample size of the calculation group was small. Therefore, the external validity of the weight ratio is unknown, even though the internal validity was considered moderate. On the other hand, the estimation method for HPF consumption in this study, which considers the percentage of HPF content for each food item in the BDHQ, may be valuable as a novel approach to minimise the misclassification of food items in dietary questionnaires. Lastly, the consumption frequency of each food in the BDHQ may have been misreported due to biases such as memory or social desirability [61], which may have resulted in misestimation of HPF intake.

## Conclusions

The results of this cross-sectional study suggest that several aspects of food choice values and food literacy were associated with HPF consumption in Japanese adults. For males, HPF consumption was positively associated with cooking skills and satiety responsiveness. For females, HPF consumption was also positively associated with satiety responsiveness while inversely associated with age, safety, and nutrition knowledge. These findings may contribute to the future development of nutritional policy to reduce HPF intake. For example, increasing nutrition knowledge may effectively reduce HPF consumption in females with low nutrition knowledge. The present study also highlights the paucity of research on this topic, suggesting the necessity and importance of further studies to clarify motives for choosing and eating HPFs. In particular, longitudinal analyses and studies considering various behavioural and sociodemographic characteristics, including marital and employment status, are needed to clarify the association of food choice values and food literacy with HPF consumption.

### Supplementary Information

**Additional file 1: Table S1.** Weight ratio (%) of HPF in 147 food codes used to calculate food intake in the BDHQ. **Figure S1.** Flow chart of food classification in the 4-d dietary record obtained from 388 Japanese adults. **Table S2.** Participant characteristics in the groups in which HPF weight proportion was calculated and validated. **Figure S2.** Bland–Altman plots for HPF consumption for (a) males and (b) females. **Text S1.** Calculation and validation of highly processed food consumption estimated from the brief diet history questionnaire.

## Data Availability

The datasets generated and analysed during the present study are not publicly available due to privacy and ethical restrictions imposed by the Ethics Committee of the University of Tokyo, Faculty of Medicine, but are available from the corresponding author upon reasonable request. The questionnaires used in this study are available from the corresponding author upon reasonable request.

## Abbreviations

AEBQ: Adult Eating Behaviour Questionnaire
BDHQ: Brief diet history questionnaire
CI: Confidence interval
DR: Dietary record
HPF: Highly processed food
JGNKQ: Japanese general nutrition knowledge questionnaire
SD: Standard deviation
