# Supplementary material for: The association of highly processed food consumption with food choice values and food literacy in Japanese adults: a nationwide cross-sectional study
Source: Int J Behav Nutr Phys Act. 2023 Dec 5;20:143. doi: 10.1186/s12966-023-01538-7 (PMC10696769; doi:10.1186/s12966-023-01538-7)
Supplement: Supplementary file 1 — Additional file 1: Table S1. Weight ratio (%) of HPF in 147 food codes used to calculate food intake in the BDHQ. Figure S1. Flow chart of food classification in the 4-d dietary record obtained from 388 Japanese adults. Table S2. Participant characteristics in the groups in which HPF weight proportion was calculated and validated. Figure S2. Bland–Altman plots for HPF consumption for (a) males and (b) females. Text S1. Calculation and validation of highly processed food consumption estimated from the brief diet history questionnaire. [file 12966_2023_1538_MOESM1_ESM.pdf]

Table S1. Weight ratio (%) of HPFs in 147 food codes used to calculate food intake in the BDHQ.

Food code[a]	Food name	Food code used for substitution[b]	Weight ratio of HPFs (%)[c]
1007	Barley, split grain, raw	---	0
1026	Common wheat, bread, white	---	100
1031	Common wheat, bread, French bread	---	100
1039	Common wheat, "Udon" (thick wheat noodles), boiled	---	7.1
1048	Common wheat, yellow alkaline noodles, boiled	---	3.8
1064	Durum wheat, macaroni and spaghetti, dry pasta, boiled	---	7.3
1083	Rice, paddy rice, non-glutinous, well-milled, raw	---	29.1
1088	Rice, short grain, paddy rice, nonglutinous rice, well-milled, "meshi" (cooked rice)	---	1.6
1128	Buckwheat, fresh noodles, boiled	---	10.3
2006	Sweet potato, tuberous root, without skin, raw	---	8.3
2010	Taro, "Satoimo", corm, raw	---	0.2
2017	Potatoes, tuber, raw	---	0.7
2022	Yam, Chinese yam, "Ichoimo", tuberous root, raw	---	0
3003	Sugars, soft sugars, white	---	32.5
4032	Soybeans, tofu, "Momen-tofu" (regular tofu)	---	0
4033	Soybeans, tofu, "Kinugoshi-tofu" (silken tofu)	---	0.7
4039	Soybeans, tofu, "Nama-age" (fried slices of drained tofu)	---	0.6
4040	Soybeans, tofu, "Abura-age" (fried thin slices of pressed tofu), uncooked	---	3.3
4041	Soybeans, tofu, "Ganmodoki" (fried mixture of crushed tofu, vegetables and ground yam)	---	100
4046	Soybeans, natto, "Itohiki-natto" (fermented whole soybean)	---	0
6038	Turnip, root, without skin, raw	---	0
6048	Pumpkin and squash, winter squash, fruit, raw	---	5.3
6061	Cabbage, common, head, raw	---	2
6065	Cucumber, fruit, raw	---	2.5
6066	Cucumber, fruit, pickles, salted pickles	---	0
6068	Cucumber, fruit, pickles, "Nukamiso-zuke" (pickled in salty rice bran paste)	---	0
6084	Edible burdock, root, raw	---	4.1
6086	Spinach mustard, "Komatsuna", leaves, raw	---	0.2
6130	Japanese radishes, Daikon, leaves, raw	---	0
6134	Japanese radishes, Daikon, root without skin, raw	---	0.9
6139	Japanese radishes, Daikon, root, pickles, "Takuan-zuke" (pickled with rice bran and salt), made of sun-dried Daikon	---	0
6148	Leaf mustard, "Takana", leaves, salted pickles	---	1.1
6153	Onions, bulb, raw	---	3.9
6160	Green bok choy, leaves, raw	---	2.4
6182	Tomatoes, fruit, raw	---	1.2
6185	Tomatoes, canned products, juice, with salt	---	0
6186	Tomatoes, canned products, tomato-based vegetable juice, with salt	---	68.4
6195	Eggplant, pickles, salted pickles	---	0
6196	Eggplant, pickles, "Nukamiso-zuke" (pickled with salty rice bran paste)	---	0
6212	Carrot, regular (European type), root with skin, raw	---	1.6
6227	Welsh onions, "Ha-negi" (large variety, green), leaves, raw	---	7.4
6231	Turnip green, "Nozawana", leaves, pickles, seasoned	---	0
6233	Chinese cabbage, head, raw	---	0.6
6235	Chinese cabbage, head, pickles, salted pickles	---	0
6267	Spinach, leaves, all season, raw	---	0
6312	Lettuce, head lettuce, crisp type, soil culture, head, raw	---	1.4
6317	East Indian lotus root, rhizome, raw	---	0.5
7012	Strawberries, raw	---	1.0
7029	Satsuma mandarins, juice sacs, normal ripening type, raw	---	0
7030	Satsuma mandarins, straight fruit juice	7149	0
7049	Japanese persimmons, nonastringent, raw	7054	1.0
7054	Kiwifruit, green flesh type, raw	---	1.0
7062	Grapefruit, white flesh type, juice sacs, raw	---	0
7063	Grapefruit, straight fruit juice	---	0
7088	Pears, sand pears, raw	---	0
7105	Citrus, "Hassaku", juice sacs, raw	---	0
7107	Bananas, raw	---	0.4
7116	Grapes, raw	---	0

Table S1. *Continued*

Food code[a]	Food name	Food code used for substitution[b]	Weight ratio of HPFs (%)[c]
7136	Peaches, raw	---	60.2
7148	Apples, without skin, raw	---	0.7
7149	Apples, straight fruit juice	---	0
8011	Mushrooms, siitake mushrooms, raw	8016	0.6
8016	Mushrooms, beech mushrooms, raw	---	0.6
9045	Algae, "Wakame", blanched and salted products, desalted	---	4.1
10006	Fish, horse mackerel, Japanese Jack mackerel, "Hirakiboshi" (salted and semi-dried split), raw	---	0
10047	Fish, sardine, Japanese pilchard, raw	---	0
10052	Fish, sardine, Japanese pilchard, "Maruboshi" (salted and dried whole), raw	---	0
10056	Fish, sardine, "Shirasuboshi" (boiled and dried whitebait), semi-dried	---	0
10067	Fish, eel, cultured, raw	10257	0
10086	Fish, skipjack tuna, caught in spring, raw	---	0
10100	Fish, righteye flounder, brown sole, raw	---	0
10134	Fish, salmon and trout, chum salmon, raw	---	6.9
10154	Fish, mackerel, chub mackerel, raw	---	0
10173	Fish, Pacific saury, with integument, raw	---	0
10180	Fish, Shishamo smelt, semi-dried, raw	---	0
10193	Fish, sea bream, red sea bream, cultured, with integument, raw	---	0
10205	Fish, cod, Pacific cod, raw	---	0.5
10218	Fish, Pacific herring, raw	10257	0
10241	Fish, yellowtail, mature, raw	---	0
10257	Fish, tuna, southern bluefin tuna, fatty meat, raw	---	0
10264	Fish, tuna, canned products, flaked white meat in oil	---	0
10281	Mollusks, short-necked clam, raw	---	0
10292	Mollusks, Pacific oyster, cultured, raw	---	0
10311	Mollusks, giant ezo-scallop, raw	---	0
10328	Crustacean, Shiba shrimp, raw	---	8.9
10335	Crustacean, snow crab, raw	10328	8.9
10345	Mollusks, Japanese common squid, raw	---	0
10361	Mollusks, common octopus, raw	---	0
11021	Beef, Japanese beef cattle, inside round, lean, raw	---	0
11032	Beef, dairy fattened steer, chuck, lean, raw	11021	0
11054	Beef, dairy fattened steer, silver side, without subcutaneous fat, raw	11021	0
11074	Beef, imported beef, flank or short plate, lean and fat, raw	---	4.1
11115	Pork, large type breed, picnic shoulder, lean and fat, raw	---	0
11117	Pork, large type breed, picnic shoulder, lean, raw	---	0
11121	Pork, large type breed, Boston butt, lean, raw	---	0
11129	Pork, large type breed, belly, lean and fat, raw	---	2.7
11166	Pork, offal and by-products, liver, raw	11232	0
11178	Pork, ham, pressed	---	100
11183	Pork, bacon	---	100
11188	Pork, sausage, dry	---	100
11190	Pork, sausage, Bologna	---	100
11191	Pork, sausage, Lyoner	11190	100
11219	Chicken, broiler, breast, meat with skin, raw	---	3.9
11220	Chicken, broiler, breast, meat without skin, raw	---	0.4
11221	Chicken, broiler, thigh, meat with skin, raw	---	5.0
11224	Chicken, broiler, thigh, meat without skin, raw	---	13.5
11232	Chicken, offal and by-products, liver, raw	---	0
12004	Eggs, hen, whole, raw	---	4.4
13003	Liquid milk, whole milk	---	6
13005	Liquid milk, containing recombined milk, low fat	---	2.8
13042	Ice cream, high fat (milk solids ≥ 15%, milk fat ≥12%)	---	100
13045	Ice cream, lacto-ice, regular (milk solids ≥ 3%, main lipid : vegetable fat)	---	100
14006	Vegetable oil, blend	---	13.3
15009	Traditional confectionery, "Kasutera" (rectangle sponge cake)	---	100
15023	Traditional confectionery, "Daifuku-mochi" (sweet rice cake stuffed with red bean paste)	---	100
15025	Traditional confectionery, "Chimaki" (bamboo-leaf-wrapped sweet rice dough, steamed)	15023	100

Table S1. *Continued*

Food code[a]	Food name	Food code used for substitution[b]	Weight ratio of HPFs (%)[c]
15031	Traditional confectionery, "Kuri-manju" (baked sweet dough stuffed with red bean paste and candied chestnuts)	---	100
15038	Traditional confectionery, "Neri-yokan" (hardened red bean agar bar)	---	100
15045	Traditional confectionery, "Karinto, black" (crunchy deep fried wheat flour dough coated with brown sugar"	---	100
15059	Traditional confectionery, "Arare" (glutinous rice cracker)	---	100
15060	Traditional confectionery, "Shoyu-senbei" (soy sauce flavored rice cracker)	---	100
15069	Bun with filling, baked bun with red bean paste filling, regular	---	100
15070	Bun with filling, baked bun with custard cream filling, regular	---	100
15073	Cake and pastry, custard cream puff	---	100
15075	Cake and pastry, layered cream cake	---	100
15076	Cake and pastry, Danish pastry	---	100
15097	Biscuits, hard biscuits	---	100
15098	Biscuits, soft biscuits	---	100
16001	Fermented alcoholic beverage, "Sake", regular	---	100
16006	Fermented alcoholic beverage, beer, pale	---	100
16010	Fermented alcoholic beverage, wine, white	---	100
16011	Fermented alcoholic beverage, wine, red	---	100
16014	Distilled alcoholic beverage, "Shochu", distilled through a continuous still	---	100
16015	Distilled alcoholic beverage, "Shochu", distilled through a pot still	---	100
16016	Distilled alcoholic beverage, whisky	---	100
16017	Distilled alcoholic beverage, brandy	---	100
16037	Green tea, "Sencha" (common grade tea), infusion	---	0
16042	Fermented tea, Oolong tea, infusion	---	0
16044	Fermented tea, black tea, infusion	---	20.6
16045	Coffee, infusion	---	1.0
16052	Carbonated beverage, fruit flavored and colored drink	---	100
16053	Carbonated beverage, cola	---	100
16054	Carbonated beverage, clear soda	---	100
17012	Edible salt, common salt, sodium chloride ≥ 99%	---	13.7
17040	Dressing, French dressing	---	100
17043	Dressing, mayonnaise, egg yolk type	---	100
17045	Miso, rice-koji miso, light yellow type	---	4.4

HPF, highly processed food; BDHQ, brief diet history questionnaire.

[a]Food codes were derived from the Standard Tables of Food Composition in Japan 2010.

[b]For items not included in the dietary record, the weight ratio of highly processed food was substituted with that of similar food.

[c]For each food code, values are calculated as a percentage of the weight of foods identified as "highly processed" in the total food consumption

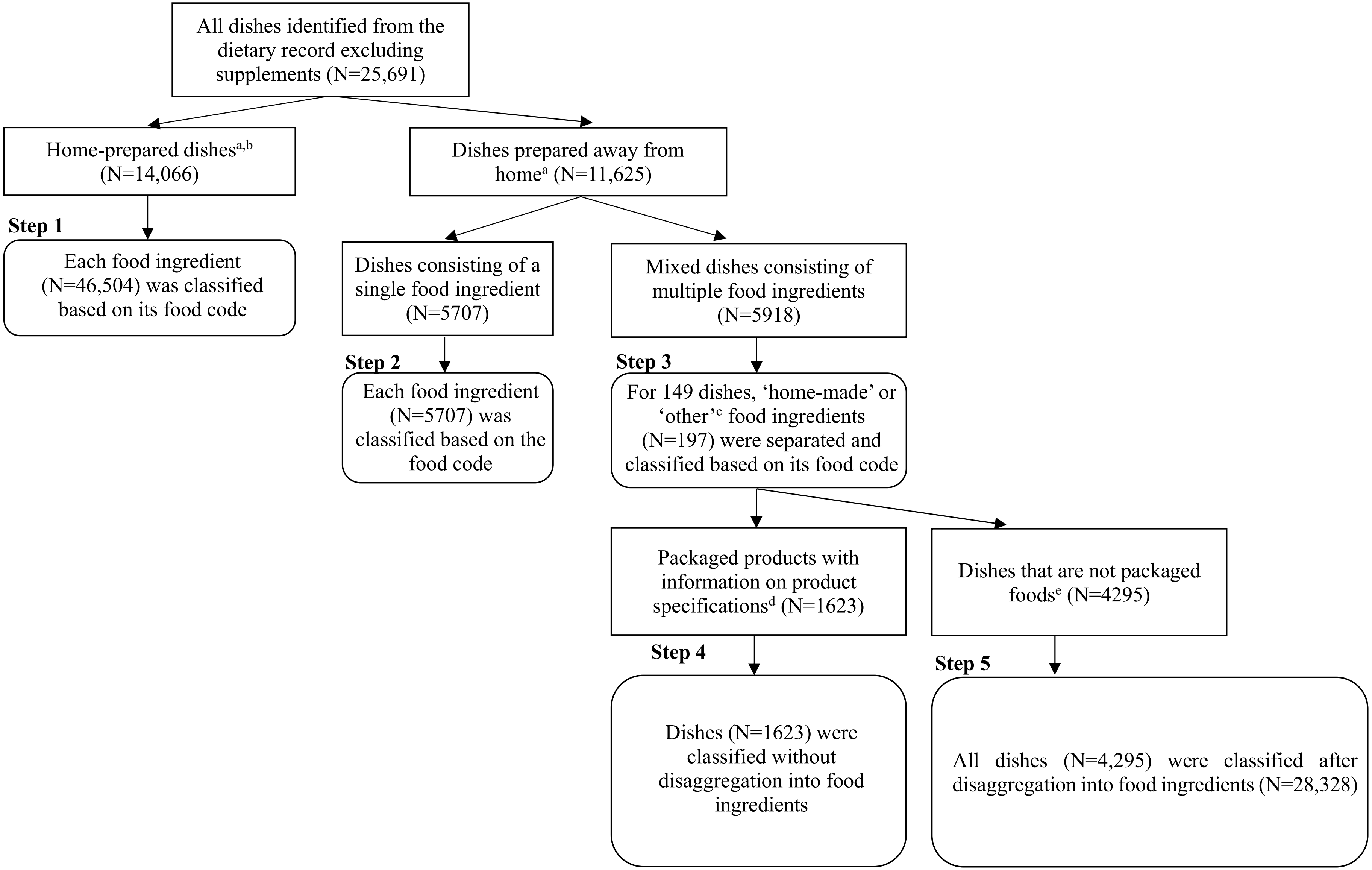

Figure S1. Flow chart of food classification in the 4-d dietary record obtained from 388 Japanese adults

[a]Self-identified by the participants.

[b]Home-prepared dishes includes foods eaten in a raw state, such as fresh fruits and vegetables.

[c]Other food ingredients included seasonings added during home cooking or used at the table at home (e.g., mayonnaise used to make sandwiches), and unprepared ingredients before cooking at home (e.g., fresh vegetables and milk), except for water.

[d]Packaged food products with the names of products, brands, or manufacturers.

[e]Dishes purchased from supermarkets, restaurant meals, and other miscellaneous dish items without information that identifies them as packaged food.

Table S2. Participant characteristics in the groups in which HPF weight proportion was calculated and validated[a].

	Male					Female				
	Calculation (N = 99)		Validation (N = 97)		*P* [b]	Calculation (N = 100)		Validation (N = 96)		*P* [b]
Age (years)	44.6	13.5	44.7	13.2	0.93	44.6	13.5	44.2	13.5	0.84
Body height (cm)	169.7	5.2	170.8	5.6	0.16	157.7	5.6	157.5	5.9	0.76
Body weight (kg)	68.7	11.0	70.6	11.7	0.23	56.3	9.5	55.9	10.6	0.78
Body mass index (kg/m^2)	23.8	3.4	24.2	3.5	0.47	22.6	3.6	22.5	3.9	0.85
Education (N, %)					0.79					0.53
Junior or senior high school	22	22.2	20	20.6		34	34.0	36	37.5	
Vocational school or junior college	30	30.3	26	26.8		48	48.0	40	41.7	
University or graduate school	47	47.5	51	52.6		18	18.0	18	18.8	
Other	0	0.0	0	0.0		0	0.0	2	2.1	
Smoking status (N, %)					0.19					0.40
Never smoker	35	35.4	31	32.0		82	82.0	72	72.0	
Past smoker	33	33.3	24	24.7		5	5.0	9	9.0	
Current smoker	31	31.3	42	43.3		13	13.0	15	15.0	
Energy intake[c] (kJ/day)	9919	2113	9781	1934	0.63	7829	1470	7967	1556	0.52
HPF comsumption[c] (g/4184 kJ)	289	178	300	169	0.68	227	111	233	116	0.67

HPF, highly processed food.

[a]Values are mean ± standard deviation, unless otherwise indicated.

[b]Differences between the two groups were analyzed using independent t-tests for age, height, weight, body mass index, and energy intake; chi-square test was used for smoking status; and Fisher's exact test for educational categories.

[c]Calculated from 4-d dietary records.

(**a**) Male (N = 97) (**b**) Female (N = 96)

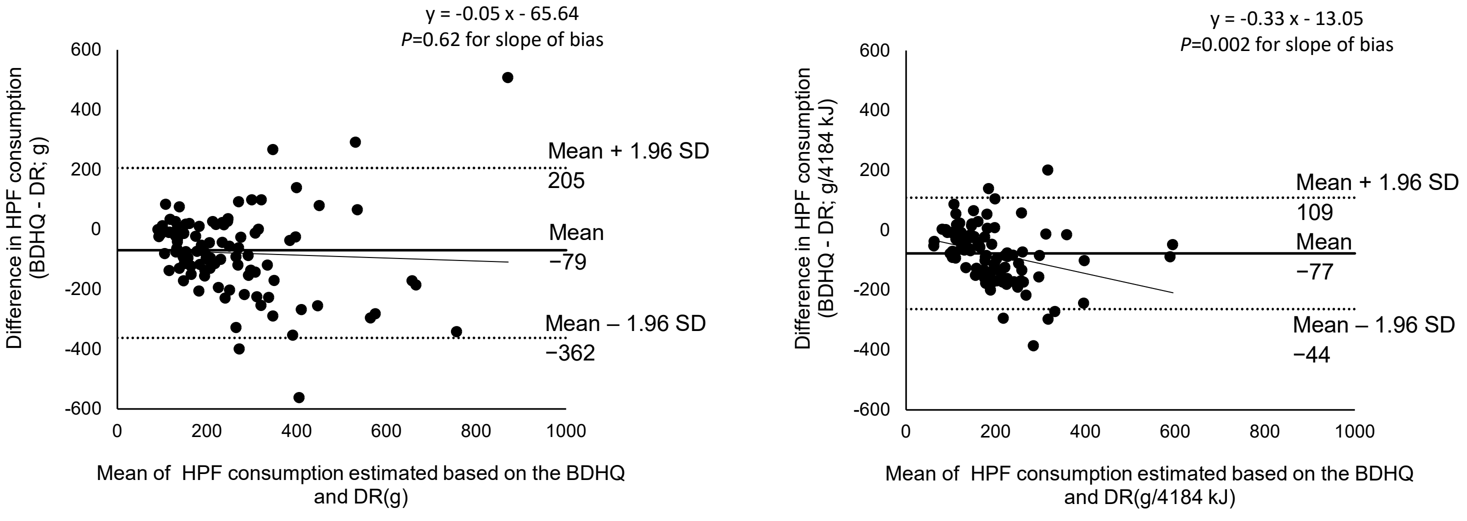

Figure S2. Bland–Altman plots for HPF consumption for (a) males and (b) females. BDHQ, brief diet history questionnaire; DR, dietary record; HPF, highly processed food; SD, standard deviation. The solid line represents the mean difference, and the dotted line represents the lower and upper limits of agreement with an added regression line.

Text S1. Calculation and validation of highly processed food consumption estimated from the brief diet history questionnaire

Dataset

The dietary record (DR) dataset used to calculate the weight ratio of highly processed food (HPF) was obtained from a nationwide survey conducted between February and March 2013 in 23 prefectures in Japan [1]. Briefly, participants were asked to record all foods and beverages consumed on four non-consecutive days (three working days and one non-working day, excluding night-shift days and days before and after a night shift). Research dietitians explained how to keep the DR on the participants and requested them to weigh foods and beverages with a digital scale (KD-812WH, Tanita, Tokyo, Japan) or measure with the spoon and cup provided. Body weight (in 0.1 kg) and height (in 0.1 cm) were measured in light clothes without shoes. Body mass index (BMI) was calculated as body weight (kg) divided by the square of height (m^2). In addition, information on sex, age, education, and smoking status (never, past, or current) was collected using a questionnaire.

The recording sheet included the following items: (i) dish names, (ii) whether dishes were prepared at home, away from home, or other (foods eaten in a raw state, such as fresh fruits and vegetables), (iii) food names (ingredients included in dishes), and (iv) approximate amounts or measured weights of foods consumed. Participants were also asked to record the names of products and manufacturers for store-bought products and the names of menus and restaurants when they dined out. In addition, participants were asked to collect packaged food packages.

Recording sheets and packages were submitted to a research dietitian at each facility immediately after recording. The research dietitians reviewed the recording sheets as soon as possible and, if necessary, asked the participants to provide additional information to clarify the name or amount of food on the sheet. The research dietitian at each facility assigned each food item a food code from the Standard Tables of Food Composition in Japan (STFCJ) [2] in a uniform procedure. For packaged foods and dishes prepared away from home, each food ingredient and its consumed weight were estimated as precisely as possible based on the approximate amount of food, the website of the restaurant or manufacturer, ingredient labels, nutrition facts labels on food packages, and cooking books.

All foods items recorded in the column ‘food names’ were then classified into three categories by the research dietitian as follows: (1) ‘home-made’: foods cooked at home (e.g., rice cooked at home and bread baked at home); (2) ‘store-bought’: ingredients of dishes prepared away from home and foods processed by the manufacturer (e.g., ready-to-heat curry, processed meat, and chocolate); and (3)

‘other’: unprocessed ingredients before cooking at home (e.g., fresh vegetables, meats, fish, and milk) or seasonings added during home cooking or used at the table at home (e.g., mayonnaise used to make sandwiches). In addition to this classification, all food codes and weights were reconfirmed by two other research dietitians at the central office of this study.

Classification of foods based on the degree of food processing in the DR

We used the framework developed by researchers at the University of North Carolina at Chapel Hill (UNC) [3] to classify foods according to the level of processing. The UNC system, developed based on the most widely used classification system, NOVA [4], provides enhanced definitions of food categories [3]. Although the UNC system classifies packaged food products with a barcode sold in the United States (US), it would be useful to classify unpackaged food products, as it provides broad and detailed examples of foods for each category, ranging from fresh vegetables (e.g., whole carrots) to refrigerated ready-made vegetable-based mixed dishes (e.g., coleslaw). A previous study showed that the UNC system had higher inter-rater reliability than the NOVA classification system [5].

According to the UNC system, the author classified all foods (except for dietary supplements) in the DR into one of the four groups:(1) unprocessed and minimally processed, (2) basic processed, (3) moderately processed, and (4) highly processed. The food classification procedure is shown below and in Figure S1.

Step 1: Home-prepared dishes (including foods eaten raw) were disaggregated into component ingredients, each of which was classified based on its food code (e.g., miso, water, spinach, and egg in miso soup cooked at home, fresh fruits, and vegetables).
Step 2: For ready-made dishes consisting of a single food ingredient, each food ingredient was classified based on its food code (e.g., black tea, black coffee, and candy).
Step 3: If dishes prepared away from home contained ‘home-made’ or ‘other’ food ingredients (mostly seasonings, such as soy sauce added to Chinese dumplings at home), that ingredient was individually classified based on its food code.
Step 4: Packaged food products (i.e., dishes prepared away from home containing multiple food ingredients with the names of products, brands, or manufacturers of packaged foods) were classified without disaggregation. For instance, we classified packaged sandwiches from convenience stores as single items.

Step 5: Other dishes prepared away from home (e.g., dishes purchased from supermarkets, restaurant meals, and other miscellaneous dish items without information identifying them as packaged food) were classified after disaggregation into food ingredients,

In Steps 1−3 and 5, each food item was classified based on the food code of the STFCJ [2], considering whether each food item was categorised as 'home-made' or 'store-bought' by the research dietitian. For instance, among tea with food code 16042 'oolong tea, infusion', those considered 'home-made' were classified into the unprocessed and minimally processed category.

Calculation of the weight ratio of HPF

To calculate the weight ratio of HPF for each code and assess the relative validity of HPF consumption estimated using them in the brief diet history questionnaire (BDHQ), we randomised participants into the calculation (N = 199) or validation (N = 193) groups using the PROC SURVEYSELECT procedure in the SAS statistical software (version 9.4, SAS Institute Inc., Cary, NC). Stratified randomisation based on age (10-year-age band) and sex was conducted to ensure that the participant's age and sex were not biased between the two datasets. No significant difference was observed between the two groups for participants' characteristics, including age, body mass index, education status, and smoking status, as well as energy intake and HPF consumption calculated from the DR (Table S2).

Using the DR data in the calculation group, the weight ratio of HPF was calculated for each food code in the BDHQ as follows: weight ratio of HPF for a food code = the sum of foods identified as 'highly processed' in the food code (g) / total food consumption in the food code (g) $\times$ 100 (Table S1). For 11 food codes not appearing in the 4-d DR in the calculation group, the weight ratio of HPF was substituted by that of similar food.

Validation of HPF consumption estimated from the BDHQ

The weight ratio of HPF was then used to calculate HPF consumption from the BDHQ in the validation group. The BDHQ computes the intake of each food group (N = 58) as the sum of the weight of food items included in the food group. For instance, the 'mayonnaise and dressing' group intake is calculated as the sum of the weights of mayonnaise (food code 17043) and French dressing (food code 17040). Therefore, we first calculated the estimated HPF consumption from each food item (e.g., mayonnaise) for each participant as the total intake of the food item (g) multiplied by its weight ratio of HPF. Subsequently, the HPF consumption from each food group (e.g., 'mayonnaise and dressing' group) was

calculated by summing the HPF consumption from each food item within that food group (e.g., mayonnaise and French dressing). Finally, the total HPF consumption was computed as the sum of the HPF consumption from all 58 food groups.

The validity of HPF consumption estimated based on the BDHQ using the weight ratio of HPF was assessed in the validation group using HPF consumption estimated from the DR as a reference. The Wilcoxon signed-rank test was used to compare the median HPF consumption (g/4184 kJ) estimated based on the BDHQ and those estimated based on the DR. The correlation between estimates from the BDHQ and DR was evaluated using Spearman correlation coefficients. In addition, a Bland–Altman plot was used to assess the agreement for estimated HPF consumption between the BDHQ and the DR [10, 11]. Statistical analyses were conducted separately for males and females, using the statistical software package SAS version 9.4. Two-sided $P < 0{\cdot}05$ was considered statistically significant.

The median (P25–P75) HPF consumption per 4184 kJ estimated based on the BDHQ was significantly lower than those estimated based on the DR in males (170.8 (117.4–260.5) g vs 249.8 (193.2–378.5) g, respectively, $P<0.0001$) and females (137.2 (105.1–177.3) g vs 218.3 (145.9–298.8) g, respectively, $P<0.0001$). The Spearman correlation coefficient for HPF consumption per 4184 kJ was 0.59 for males (N = 97) and 0.46 for females (N = 96), indicating moderate correlation. The limits of agreement were wide for both sexes, mainly because of increased dispersion with larger serving sizes. The Bland–Altman plots are shown in Figure S2. The limits of agreement were wide for both sexes, mainly because of increased dispersion with larger HPF consumption. Linear regression analysis demonstrated that the slope of the mean bias for each food item was significantly different from 0 in females, indicating a proportional bias such that differences between the true and estimated serving size increased as the mean HPF consumption increased.

References

1. Asakura K, Uechi K, Masayasu S, Sasaki S. Sodium sources in the Japanese diet: difference between generations and sexes. Public Health Nutr. 2016;19:2011-23. doi: 10.1017/S1368980015003249.
2. Council for Science and Technology; Ministry of Education & Culture S, Science and Technology, Japan. Standard Tables of Food Composition in Japan, 2010. Official Gazette Co-Operation of Japan; 2010. (in Japanese)
3. Poti JM, Mendez MA, Ng SW, Popkin BM. Is the degree of food processing and convenience linked with the nutritional quality of foods purchased by US households? Am J Clin Nutr. 2015;101:1251-62. doi: 10.3945/ajcn.114.100925.

4. Monteiro CA, Cannon G, Levy RB, et al. Ultra-processed foods: what they are and how to identify them. Public Health Nutr. 2019;22:936-941. doi:10.1017/S1368980018003762
5. Bleiweiss-Sande R, Chui K, Evans EW, Goldberg J, Amin S, Sacheck J. Robustness of food processing classification systems. Nutrients. 2019;11:1344. doi: 10.3390/nu11061344.
6. Food and Agriculture Organization of the United Nations. Guidelines on the collection of information on food processing through food consumption surveys. https://www.fao.org/3/i4690e/I4690E.pdf. 2015. Accessed June 2021.
7. Liu J, Steele EM, Li Y, Karageorgou D, Micha R, Monteiro CA, et al. Consumption of ultraprocessed foods and diet quality among U.S. Children and adults. Am J Prev Med. 2022;62:252-64. doi: 10.1016/j.amepre.2021.08.014.
8. Martínez Steele E, Popkin BM, Swinburn B, Monteiro CA. The share of ultra-processed foods and the overall nutritional quality of diets in the US: evidence from a nationally representative cross-sectional study. Popul Health Metr. 2017;15:6. doi:10.1186/s12963-017-0119-3
9. Louzada MLDC, Ricardo CZ, Steele EM, Levy RB, Cannon G, Monteiro CA. The share of ultra-processed foods determines the overall nutritional quality of diets in Brazil. Public Health Nutr. 2018;21:94-102. doi:10.1017/S1368980017001434
10. Bland J, Altman D. Statistical methods for assessing agreement between two methods of clinical measurement. Lancet. 1986;1:307-10.
11. Montenij LJ, Buhre WF, Jansen JR, Kruitwagen CL, de Waal EE. Methodology of method comparison studies evaluating the validity of cardiac output monitors: a stepwise approach and checklist. Br J Anaesth. 2016;116:750-8. doi: 10.1093/bja/aew094.
